# Surface Morphology Evolution Mechanisms of InGaN/GaN Multiple Quantum Wells with Mixture N_2_/H_2_-Grown GaN Barrier

**DOI:** 10.1186/s11671-017-2115-8

**Published:** 2017-05-16

**Authors:** Xiaorun Zhou, Taiping Lu, Yadan Zhu, Guangzhou Zhao, Hailiang Dong, Zhigang Jia, Yongzhen Yang, Yongkang Chen, Bingshe Xu

**Affiliations:** 1 Key Laboratory of Interface Science and Engineering in Advanced Materials, Ministry of Education, Taiyuan University of Technology, Taiyuan, 030024 China; 2 Research Center of Advanced Materials Science and Technology, Taiyuan University of Technology, Taiyuan, 030024 China

**Keywords:** GaN barrier, Hydrogen, Surface, Interface

## Abstract

Surface morphology evolution mechanisms of InGaN/GaN multiple quantum wells (MQWs) during GaN barrier growth with different hydrogen (H_2_) percentages have been systematically studied. Ga surface-diffusion rate, stress relaxation, and H_2_ etching effect are found to be the main affecting factors of the surface evolution. As the percentage of H_2_ increases from 0 to 6.25%, Ga surface-diffusion rate and the etch effect are gradually enhanced, which is beneficial to obtaining a smooth surface with low pits density. As the H_2_ proportion further increases, stress relaxation and H_2_ over- etching effect begin to be the dominant factors, which degrade surface quality. Furthermore, the effects of surface evolution on the interface and optical properties of InGaN/GaN MQWs are also profoundly discussed. The comprehensive study on the surface evolution mechanisms herein provides both technical and theoretical support for the fabrication of high-quality InGaN/GaN heterostructures.

## Background

InGaN/GaN-based high-brightness light-emitting diodes (LEDs) and laser diodes, as the representative devices of III-nitrides, have attracted much attention owing to their important role in digital signage, high-density optical storage, and general illumination [1–10]. Generally speaking, fabrication of blue or green LEDs requires relatively high indium composition of InGaN layer [11, 12]. Although the reduction of growth temperature and the increase of growth rate of the quantum well (QW) can alleviate indium atom desorption to obtain high indium content, these methods also deteriorate the optical performance of InGaN/GaN multiple quantum wells (MQWs) by worsening interface abruptness and introducing more defects [13, 14]. Moreover, these defects usually act as nonradiative recombination centers, thus weakening the internal quantum efficiency of the device [15–19]. Therefore, achieving required indium content while maintaining high material quality is still a big challenge.

In order to settle the problems mentioned above, various growth techniques have been employed in striving for smooth morphology and sharp interfaces within the InGaN/GaN stack. Quantum barriers (QBs) grown at elevated temperature [20, 21] and growth interruption after QWs [12, 22] are widely used to improve the morphology of InGaN/GaN heterostructures. However, they all have their own limitations. For instance, barriers grown at high temperature may lead to severe In loss [14, 23]. Although growth interruption can improve morphology as well as reduce inclusions, it is at the expense of the optical quality of the QWs [21]. Recently, it is reported that introducing a small amount of hydrogen during the growth of GaN barriers can improve both optical and interface properties [24–28]. However, the effect mechanism of H_2_ on surface evolution of InGaN/GaN MQWs has not been fully understood yet.

In this paper, the effects of H_2_ proportion, defined as H_2_ flow divided by total carrier gas flow, during GaN barrier deposition, on surface morphology evolution are systematically investigated. Ga surface-diffusion rate, stress relaxation, and H_2_ etching effect are suggested to be the three main factors, affecting surface evolution. The dominant factors and their influences on the surface evolution are comprehensively discussed, which provides a technical guideline to obtain high-quality InGaN/GaN heterostructures.

## Methods

The InGaN/GaN MQW structures were grown on c-plane sapphire substrate by Aixtron TS300 metal organic chemical vapor deposition system. Trimethylgallium (TMG), triethylgallium (TEG), trimethylindium (TMI), and ammonia (NH_3_) were used as precursors. Silane (SiH_4_) was used as the n-type dopant source. The structure was composed of 3.2-μm-thick undoped GaN layer and nominally six-period 2.4-nm-thick InGaN QWs separated by 11-nm-thick lightly Si-doped (n-doping = 3×10^17^cm^−3^) GaN barriers. A 1.0-nm-thick low temperature GaN cap layer (LT-GaN) was deposited immediately after the growth of QW layer. InGaN wells and GaN barriers were grown at 730 and 850 °C, respectively. A conventional InGaN/GaN MQWs sample, labeled as S_1_, was grown in nitrogen atmosphere. Four other samples, denoted as S_2_, S_3_, S_4_, and S_5_, were grown with different proportion of H_2_ flow to total carrier gas (N_2_ + H_2_) during barriers deposition, with the other growth parameters the same with S_1_. The percentage of H_2_ was 2.5% (S_2_), 6.25% (S_3_), 10% (S_4_), and 50% (S_5_), respectively.

The structures of InGaN/GaN MQWs were characterized by PANalytical Empyrean high resolution x-ray diffraction (HRXRD) system. Surface morphology was obtained by atomic force microscopy (AFM) (SPA-300HV) using tapping model. Room temperature (RT) photoluminescence (PL) properties of the samples were studied by 226-nm Nd-YAG laser with an excitation power density of 1.36 W/cm^2^.

## Results and Discussion

**Fig. 1:**
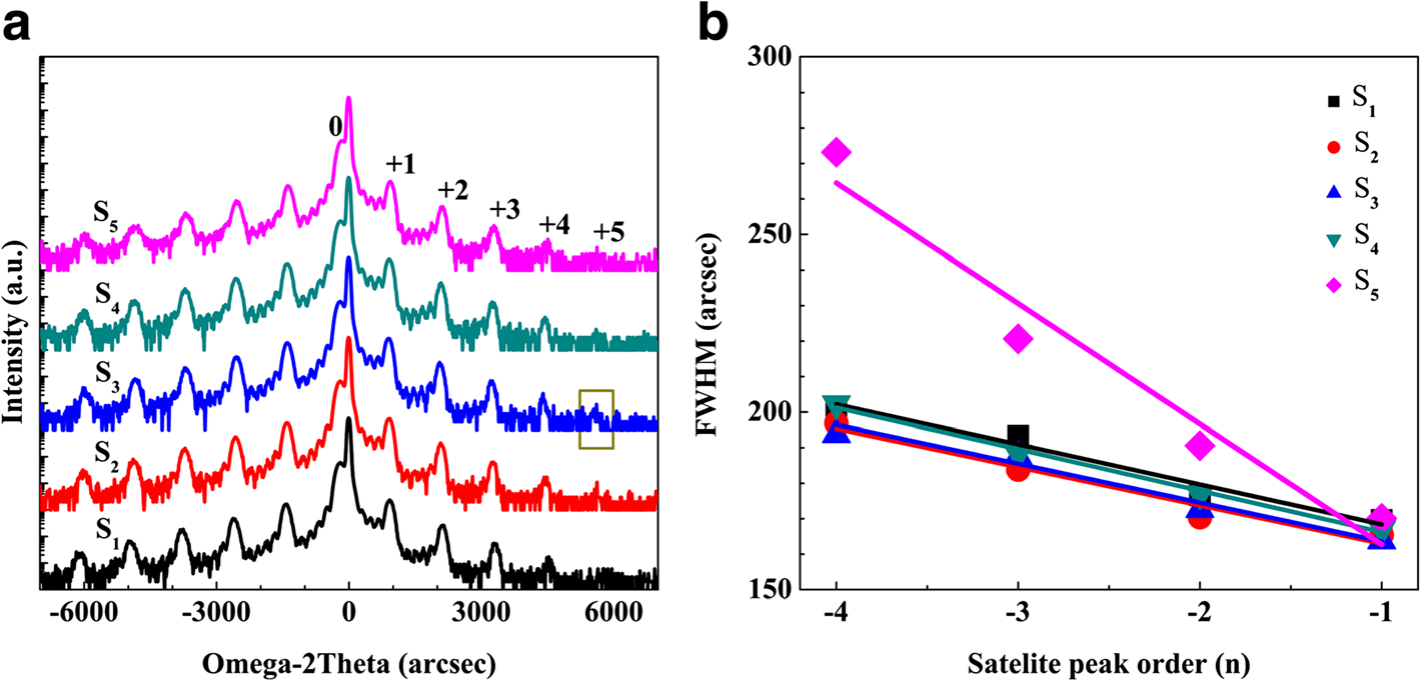
**a** The HRXRD ω-2θ scanning results of S_1_–S_5_. **b** The FWHM as a function of the satellite peak order and its linear fitting for the five samples

The HRXRD ω-2θ scanning results of S_1_–S_5_ are illustrated in Fig. 1a. The strongest peak located at the center belongs to the underlying GaN template, and the satellite peaks correspond to the periodicity of the MQWs. It is found that the full-width at half-maximum (FWHM) of the strongest peaks in all samples is almost the same, indicating the similar crystal quality of GaN buffer layers for all samples. The presence of clearly distinguished “ + 4th” diffraction peak in samples S_2_–S_4_ manifest the improvement of crystal quality under low H_2_ percentage. The appearance of the “ + 5th” diffraction peak (represented by the rectangle in Fig. 1a) and the minimum FWHM value of InGaN “−1st” diffraction peak indicate the best interface quality in sample S_3_. The structure parameters determined by fitting the measured XRD curves are summarized in Table 1. The period thicknesses of the five samples are almost the same, and the values keep around 14.4 nm. The indium contents of the InGaN wells for samples S_1_ to S_4_ keep around 11.8%, while the value drops to 9.9% for S_5_. A large amount of H_2_ may etch the GaN LT-cap layer and then react with indium atoms in QWs, which result in the reduction of average indium content [29]. The roughness of the interface can be calculated by fitting FWHM of the XRD satellite peak by the following equation [26, 30]:

**Table 1:** Structure parameters of InGaN/GaN MQWs determined by HRXRD fitting

Sample	H_2_ percentage (%)	In content (%)	FWHM of InGaN “−1st” diffraction peak (arcsec)	Slope of liner fitting
S_1_	0	11.84	169.66	−11.27
S_2_	2.50	12.04	165.43	−10.79
S_3_	6.25	11.67	163.77	−10.49
S_4_	10	11.60	167.19	−11.68
S_5_	50	9.90	170.13	−33.92

1 $$ \varDelta {\omega}_{\mathrm{n}}=\varDelta {\omega}_0+\left[{\left( \ln 2\right)}^{1/2}\cdot \kern0.3em \varDelta {\theta}_{\mathrm{M}}\cdot \frac{\gamma}{D}\right]\cdot n $$

where *Δω*_n_ represents the FWHM of the n-th satellite peak, *Δω*_0_ is the intrinsic width of satellite peaks, *Δθ*_*M*_ is the angle spacing between the adjacent satellite peaks, *D* is the period thickness of the InGaN/GaN MQW and *γ* is the interface roughness. Figure 1b shows the linear relationship between FWHMs and satellite peak orders. The slope of the fitting line is related to the QW/QB interface roughness. The fitting results show that interface roughness is gradually reduced as the H_2_ percentage increases, and the optimum value is achieved at 6.25% of H_2_ (S_3_), as shown in Table 1. With further raising in the percentage to 50% (S_5_), the interface roughness is increased dramatically. Hence, the ratio of H_2_ during barrier growth has great impact on interface quality. A small percentage (0–6.25%) of H_2_ is favorable to obtaining sharp interface, while a large amount of H_2_ (50%) seriously roughens the interface.

**Fig. 2:**
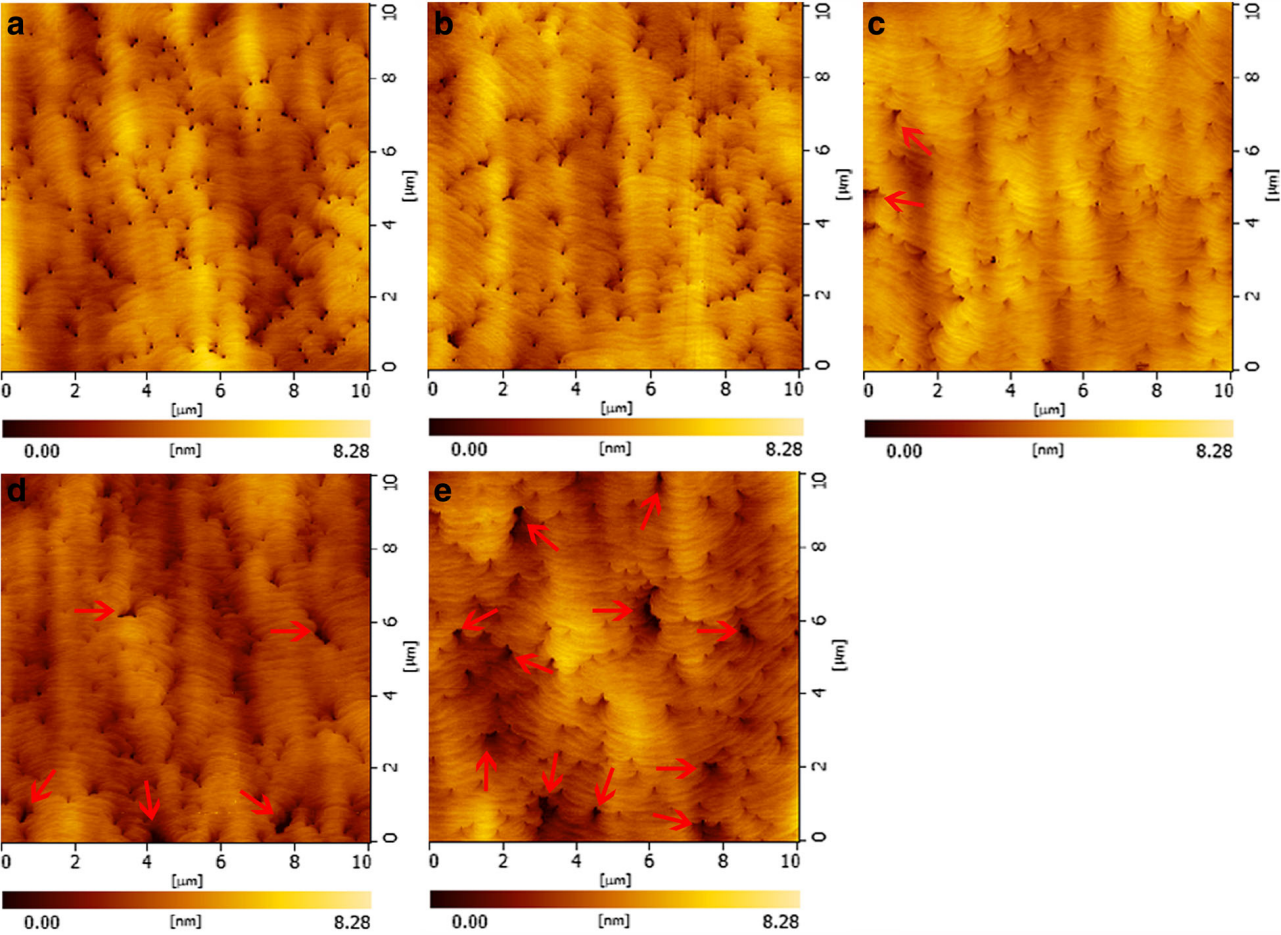
The AFM images (10 × 10 μm) of five samples: **a** S_1_, **b** S_2_, **c** S_3_, **d** S_4_, and **e** S_5_

**Fig. 3:**
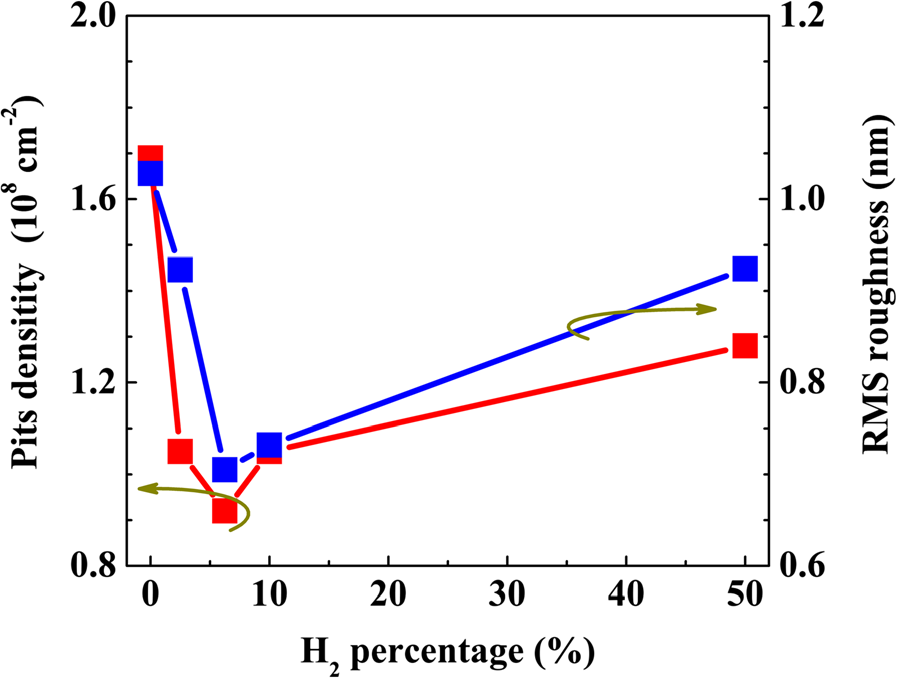
The variation trend of pits density and RMS surface roughness under different H_2_ percentage in carrier gas during the growth of barriers

The AFM images of sample S_1_–S_5_ are shown in Fig. 2a–e. The dark points are mainly V-pits [14, 31], which initiate at the threading dislocations (TDs) [21, 27]. The root mean square (RMS) surface roughness under different H_2_ percentage is illustrated in Fig. 3. The reference sample S_1_ grown with H_2_-free condition possesses the coarsest surface with an RMS roughness of 1.028 nm. The RMS value decreases with the increase of H_2_ percentage, and achieves the minimum value (0.705 nm) at 6.25% of H_2_, as shown in Fig. 3. As the H_2_ percentage raises to 10%, the surface gets slightly rougher. With further increase in H_2_ percentage to 50%, many large holes are formed, as pointed out by red arrows in Fig. 2, and surface RMS roughness dramatically increases to 0.924 nm.

**Fig. 4:**
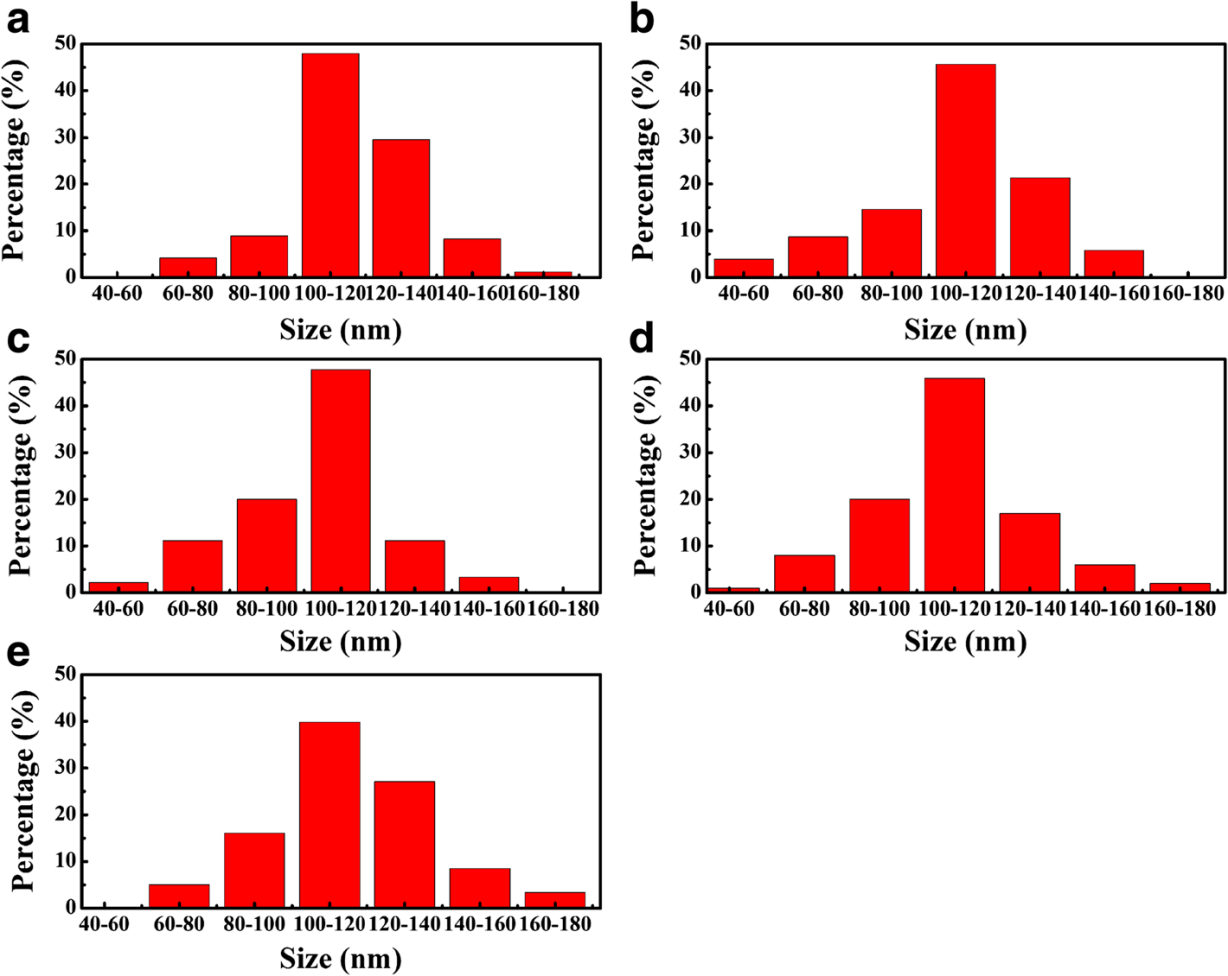
The distribution of pit size for the five samples: **a** S_1_, **b** S_2_, **c** S_3_, **d** S_4_, and **e** S_5_

Figure 4 shows the statistical calculated diagram of pits size distributions for the five samples. It can be seen that as 2.5% H_2_ is introduced, the smallest pits (<60 nm) start to emerge, and the largest pits (>160 nm) disappear. As H_2_ percentage increases to 6.25% (S_3_), the proportion of pits at size 80–100 nm is significantly raised, and that of large pits (>140 nm) is dramatically reduced to the minimum value. With further increase in the H_2_ percentage to 10%, the largest pits begin to emerge again. When 50% H_2_ is introduced, the ratio of large pits is dramatically increased. Hence, the pits size can be reduced by introducing a small amount of H_2_, and the optimum value is acquired at 6.25% percentage. However, the pits size shows an increase trend as H_2_ percentage further rises.

It is obvious that the evolution trend of RMS surface roughness is highly consistent with that of pits size, which may relate to the growth mode affected by the formed pits. Once the pits are formed, indium atoms will first nucleate at the point where the TDs intersect the InGaN/GaN interface [32–35], then island growth starts and finally island growth mode transfers to 2-Dimensional growth. In other words, the presence of V-pits will delay the 2-dimensional growth, then roughen the surface. The larger the size is, the more obvious the delay can be.

In order to elucidate the surface evolution mechanism under different H_2_ percentages, the variation trend of V-pits size is discussed in detail. As H_2_ percentage increases from 0 to 6.25%, the decrease in V-pits size possibly comes from the following two parts. First, the formed Ga-H complex may enhance the incorporation efficiency of Ga atoms on $\left\{10\overline{1}1\right\}$ plane [35]. It is reported that the adsorption energy of the Ga-H complex is about 1.2 eV smaller than that of single Ga adatoms [28]. Hence, the attachment of hydrogen to Ga adatom could significantly weaken the bond to the surface, which benefits the surface diffusion of Ga atoms [28, 36]. Another important reason is the gradually enhanced etching effect with the increase of H_2_ percentage. Shiojiri et al. reported that indium atoms can be easily trapped and segregated around the core of TDs, which plays a role of a small mask that hinders Ga atoms migration [37]. Hence, introducing H_2_ during the growth can effectively eliminate indium-rich clusters at InGaN/GaN interface, and contribute to surface migration of Ga atoms [37–39]. In addition, hydrogen can etch some unstable areas, such as dislocation sites and V-pits [40–43]. It is reported that dislocation sites are unstable due to the high strain energy and weak binding energy, and these sites can be easily dissociated during the etching process [41–43]. Moreover, the V-pit commonly consists of six symmetric N-terminated $\left\{10\overline{1}1\right\}$ facets [44, 45], which is much weaker during the etching process as compared with Ga-terminated facets [42, 43]. Therefore, when H_2_ arrives at the surface, it is difficult to etch most of the GaN on the surface due to the high stability of Ga-face. Thus, H_2_ etching occurs mainly at dislocation sites and V-pits [42, 43], causing the decomposition of GaN. Due to the low growth temperature of GaN barrier, the decomposition effect of GaN is weak when hydrogen percentage is low [26]. Hence, the enhanced Ga atoms incorporation plays a dominate role in surface evolution, which is beneficial in reducing the size and density of pits and in turn enhances the 2-Dimensional growth and suppresses the formation of new pits, and finally conductive to smooth surface. The correlation between pits density and H_2_ percentage is presented in Fig. 3. It is shown that the highest pit density (1.69 × 10^8^ cm^−2^) exists in the H_2_-free sample. While a small amount of H_2_ is added in the carrier gas, pits density is gradually reduced and reaches the lowest value (0.92 × 10^8^ cm^−2^) in sample S_3_. With further increase in H_2_ proportion to 50%, pits density is significantly increased to 1.28 × 10^8^ cm^−2^. These results indicate that adding a little H_2_ in the growth of barrier layer can suppress the formation of new pits. However, the suppression of new pits formation could lead to strain accumulation inside the layer, and the strain may relax via formation of new dislocations and other defects such as big pits in S_4_ and S_5_ [21], which will deteriorate the quality of the surface, as well as the InGaN/GaN interface.

**Fig. 5:**
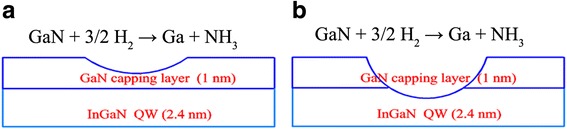
**a** The etch effect on LT-GaN cap layer with H_2_ percentage lower than 10%. **b** the H_2_ over etch effect on LT-GaN capping layer under large H_2_ percentage

It is worth to mention that the large holes (>200 nm) as marked with red arrows do not appear in samples S_1_ and S_2_, and they only start to appear as H_2_ percentage becomes larger than 2.5%. The hole size in S_5_ is much larger than that in samples S_3_ and S_4_, which may relate to the following two possible mechanisms about hydrogen over-etching mechanisms. One is the hydrogen over-etching on dislocation sites and V-pits. As aforementioned, the enhanced diffusion of Ga atoms plays a dominant role when hydrogen percentage is low. However, this leading role shifts to the enhanced GaN decomposition around dislocation sites and V-pits when large amounts of hydrogen are applied. The hydrogen can diffuse along the dislocation line and then etch the surrounded unstable sites both vertically and longitudinally, which could decrease the average indium contents in MQWs region, degrade well/barrier interface quality and also form large holes on the surface. Another possible mechanism is about hydrogen over-etching on LT-GaN cap layer. As H_2_ proportion lower than 2.5%, the H_2_ etch effect on LT-GaN cap layer is negligible. As the H_2_ percentage increases to 10%, the H_2_ etch effect on LT-GaN cap is illustrated in Fig. 5a. H_2_ only etches a part of the cap layer, which has little influence on the QW layer, as evidenced by almost unchanged indium contents, and positive influence on the surface morphology, as confirmed by low pits density and small size of the holes. However, under large H_2_ percentage, the LT-GaN cap layer may be partly etched away and QW layer be directly exposed to H_2_, as presented in Fig. 5b. Under this case, H_2_ will react with indium atoms in QW layer, leading to significant indium loss, large size, and high-density holes, and consequently dramatically deteriorates InGaN/GaN interface and surface qualities. Hence, surface morphology evolution is an integrated effect of surface diffusion rate, strain relaxation and H_2_ etch effect. For sample S_2_ and S_3_ with H_2_ percentage lower than 6.25%, gradually enhanced surface diffusion rate and H_2_ etch effect play a dominant role, which contributes to smoother surface and lower pits density. With further increase in the percentage to 10% (S_4_), surface properties become slightly worse as a result of stress relaxation. The surface morphology of sample S_5_ grown in 50% H_2_ is mainly controlled by H_2_ over-etching effect and strain relaxation in InGaN QWs, which leads to many large holes and the worst surface.

**Fig. 6:**
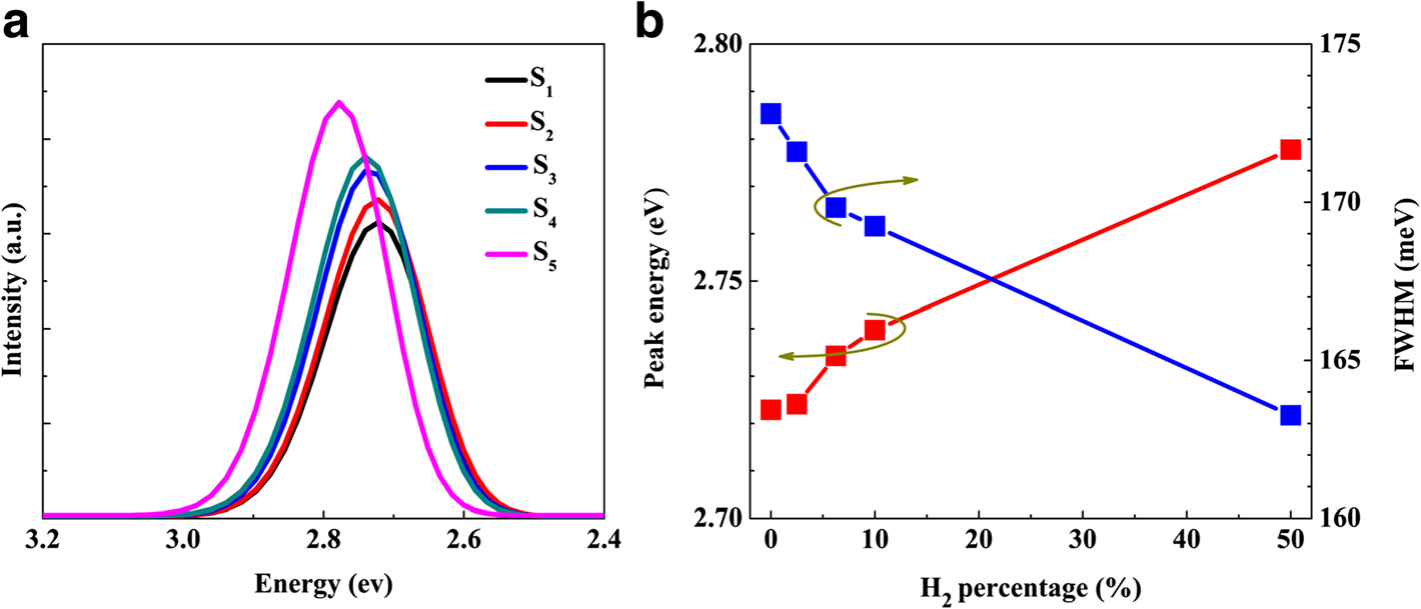
**a** The measured room temperature PL spectra of the samples. **b** Gaussian-fitted FWHM and peak energy versus H_2_ percentage in carrier gas

Figure 6a shows the measured room temperature PL spectra of the five samples. It can be seen that the PL intensity shows an increase trend and peak energy exhibits blue-shift as H_2_ percentage increases. Compared with that of sample S_1_ without H_2_, the integrated PL intensity of samples S_2_–S_5_ is increased by 7.0, 15.8, 19.3, and 31.6%, respectively. For samples S_2_–S_4_, slightly blue-shifted peak energy and reduced FWHM are observed, as shown in Fig. 6b. As aforementioned, the structure parameters of sample S_2_–S_4_ are quite similar. Hence, the slightly changed spectral characteristics along with enhanced PL intensity are mainly caused by the enhanced surface and interface quality, and the partial relaxation of stress in QWs alleviating quantum confined stark effect (QCSE) [21, 46]. In contrast, the significantly reduced FWHM, blue-shifted peak energy and enhanced PL intensity of sample S_5_ may result from strain relaxation and the lowest indium content caused by H_2_ over-etching effect, both of which can greatly alleviate QCSE effect in MQWs [46–49]. In addition, H_2_ can eliminate impurities such as carbon and oxygen in active region, which would benefit the improvement of the PL intensity [50, 51].

## Conclusions

In summary, the effect of H_2_ percentage during the barriers growth on InGaN/GaN MQWs properties has been systematically studied. As a small percentage of H_2_ (≤6.25%) is introduced, the combined effect of enhanced H_2_ etch effect and surface diffusion contribute to the improvement of surface, interface and optical properties. In spite of the strongest PL intensity achieved by introducing large percentage H_2_ (50%), the integrated effect of H_2_ over-etching and stress relaxation degrades surface and interface quality of the InGaN/GaN MQWs. Hence, the use of H_2_ with appropriate proportion during the barriers growth can achieve smooth surface with low pits density and enhanced optical performance. The profound discussions of surface evolution mechanism here clearly depict the physical pictures of surface evolution process under different growth conditions, which is helpful for the fabrication of high-quality GaN-based devices.

## Abbreviations

AFM: Atomic force microscopy
HRXRD: High-resolution x-ray diffraction
LED: Light-emitting diode
RMS: Root mean square
PL: Photoluminescence
